# Dispersed and Co-Continuous Morphologies of Epoxy Asphalt Bond Coats and Their Effects on Mechanical Performance

**DOI:** 10.3390/molecules30173513

**Published:** 2025-08-27

**Authors:** Suzhou Cao, Haocheng Yang, Xinpeng Cui, Zhonghua Xi, Jun Cai, Junsheng Zhang, Hongfeng Xie

**Affiliations:** 1 MOE Key Laboratory of High Performance Polymer Materials and Technology, School of Chemistry and Chemical Engineering, Nanjing University, Nanjing 210023, China; 522024240001@smail.nju.edu.cn (S.C.); 522023240043@smail.nju.edu.cn (H.Y.); 15299908527@163.com (X.C.); 2 Experimental Chemistry Teaching Center, School of Chemistry and Chemical Engineering, Nanjing University, Nanjing 210023, China; xizh@nju.edu.cn; 3 Public Instrument Center, School of Chemistry and Chemical Engineering, Nanjing University, Nanjing 210023, China; caijun@nju.edu.cn

**Keywords:** epoxy asphalt, bond coat, phase separation, phase inversion, co-continuous structure

## Abstract

The co-continuous microstructure represents an ideal configuration for polymer-modified asphalts. Consequently, determining the optimum polymer content hinges on establishing this critical network between polymer and bitumen. In this study, epoxy asphalt bond coats (EABCs) exhibiting three distinct morphologies (epoxy-dispersed, co-continuous, and bitumen-dispersed) were prepared. Phase structure evolution and the final cured morphology were analyzed using a laser scanning confocal microscope (LSCM). Rotational viscosity–time characteristics, tensile properties, single-lap shear strength, and pull-off adhesion strength were characterized using various techniques. Results indicated that the viscosity of EABCs at the late stage of the curing reaction increased with increasing epoxy resin (ER) concentration, whereas the time required for EABCs to reach a viscosity of 5 Pa·s decreased. LSCM analysis revealed that EABCs exhibited three distinct morphologies dependent on ER concentration: (1) a bitumen-continuous morphology with dispersed epoxy domains (41–42 vol.% ER) formed via a nucleation and growth mechanism; (2) a co-continuous structure (43–45 vol.% ER); and (3) an epoxy-continuous structure with dispersed bitumen domains (46 vol.% ER). Furthermore, the EABC with 42 vol.% exhibited a transitional morphology between bitumen-continuous and co-continuous structures. A significant improvement in mechanical properties occurred during the transition from the bitumen-continuous (41 vol.% ER) to the co-continuous morphology (43 vol.% ER): tensile strength, elongation at break, and toughness increased by 524%, 1298%, and 2732%, respectively. Simultaneously, pull-off adhesion strength and single-lap shear strength rose by 61% and 99%, respectively. In contrast, mechanical properties increased only gradually during the co-continuous phase and the subsequent transition to an epoxy-continuous morphology (45–46 vol.% ER). Considering cost, rotational viscosity–time dependence, and mechanical performance, an ER concentration of 43 vol.% (within the co-continuous region) is optimal for EABC production.

## 1. Introduction

Epoxy asphalts have been extensively employed in orthotropic steel deck bridges since 1967 due to their superior adhesion, enhanced stability, durable skid resistance, reduced permeability, and excellent performance across low and high temperatures [1,2,3]. Based on their applications, epoxy asphalts are categorized into two types: binders for mixture production and bond/tack coats for adhering to steel decks or overlays while providing waterproof protection [4,5,6]. Depending on operating temperatures, epoxy asphalt binders (EABs) can be further classified into cold-mix (CEAB, ambient temperature), warm-mix (WEAB, 110–130 °C), and hot-mix (HEAB, 160–190 °C) systems [7,8]. Epoxy asphalt binders are typically paired with epoxy asphalt bond coats (EABCs) or epoxy resin (ER) bond coats (ERBC) in pavement systems [9,10,11]. For instance, WEAB employs EABCs, while HEAB utilizes ERBCs [12,13].

Epoxy asphalts consist primarily of epoxy resin and bitumen. When mixed, chemical reactions occur between epoxide groups in epoxy monomers/oligomers and active hydrogen atoms of curing agents, forming crosslinked networks within the material. This increases the molecular weight of the epoxy polymer, which reduces its solubility in the bitumen and thereby induces phase separation [14]. Additionally, the phase structures of epoxy asphalts are determined by the epoxy resin-to-bitumen ratio [15,16,17,18]. Regarding domain morphology, the minor phase disperses in the major continuous phase, with domain size distribution varying according to the composition of epoxy resin/bitumen components. Typically, the domain size of the dispersed component increases with its concentration due to coalescence, with phase inversion occurring beyond a particular compositional threshold [19].

The properties of epoxy asphalts are directly determined by their morphology [20,21,22]. Given the substantial mechanical performance difference between cured epoxy resin and bitumen, high-performance epoxy asphalt applications (such as orthotropic steel deck bridges and open-graded friction courses) typically require epoxy-dominated phases with dispersed bitumen domains [23,24]. Unlike thermoplastic polymer modifiers, cured epoxy resin exhibits no maltene-absorption capability, preventing swelling in bitumen [25]. Consequently, polymer-dominated epoxy asphalts require significantly higher epoxy resin content (approximately 40 wt%) compared to thermoplastic polymer-modified asphalts (5–7 wt%) [26,27,28].

Phase inversion represents a critical threshold for the mechanical properties of epoxy asphalts. A dramatic enhancement in mechanical performance occurs when the phase structure transitions from bitumen-dominated to epoxy-dominated [29,30]. Liu et al. [29] demonstrated this phenomenon quantitatively: during phase inversion (at epoxy contents between 40 and 50 wt%), HEAB exhibits substantial property improvements—tensile strength increases from 2.1 to 5.7 MPa, elongation at break from 142 to 164%, toughness from 4.0 to 5.1 MJ/m^3^, and Young’s modulus from 27 to 41 MPa.

Notably, a co-continuous (or bi-continuous) structure forms in immiscible polymer blends near the phase inversion composition (phase inversion point), marking the transition between two distinct dispersed morphologies [31,32,33]. In classical terms, an ideal co-continuous structure is characterized by the simultaneous presence of two interpenetrating continuous phases within the same volume, indicating that both components maintain three-dimensional spatial continuity at a specific mixing scale [34]. According to percolation threshold theory, while recognizing that real-world structures represent a spectrum of morphological types rather than perfect networks, a co-continuous structure can be pragmatically defined as follows: each phase must contain at least one coherent continuous pathway that percolates through the entire volume [35]. This definition accommodates the possible presence of isolated, dispersed domains that are not integrated into the network structure. The critical volume fraction (percolation threshold) marks the transitional composition where the system evolves from containing exclusively dispersed domains of one phase to developing an infinite network structure together with residual dispersed domains. Compared to dispersed morphologies, co-continuous structures attract greater scientific interest due to their capacity to synergistically combine the optimal properties of both constituent polymers [36,37,38].

In polymer-modified asphalts (PMAs), co-continuous structures (two interlocked continuous phases) develop alongside dispersed morphologies [39,40,41,42]. These structures drastically improve properties, such as rutting resistance and elasticity, compared to conventional PMAs. The polymer concentration enabling this critical network formation determines the optimal content [43], establishing co-continuous morphology as the preferred phase structure for balancing PMA performance and cost [44,45,46].

Although dispersed and phase-inverted morphologies of epoxy asphalts have been well-documented, co-continuous structures remain unexplored. Given epoxy resin’s high cost and required dosage compared to conventional thermoplastic polymers, optimizing the concentration for co-continuous epoxy asphalts is critical.

**Figure 1:**
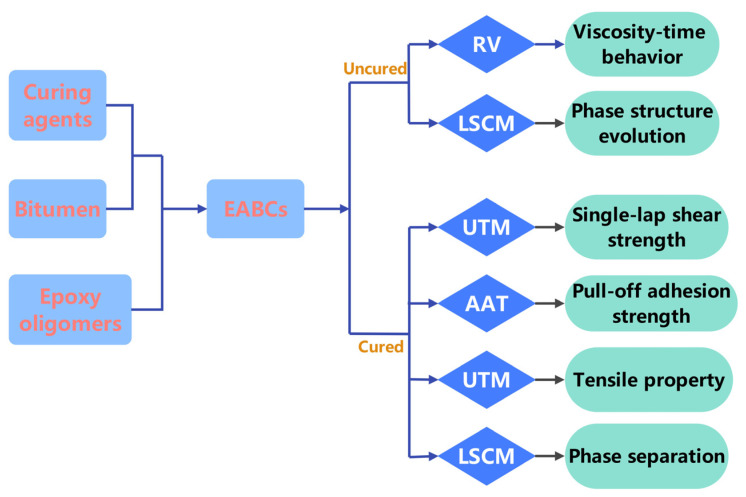
Schematic diagram of EABC preparation and characterization processes.

We engineered epoxy asphalt bond coats with three distinct phase structures: bitumen-continuous, co-continuous, and epoxy-continuous. A laser scanning confocal microscope (LSCM) tracked structural evolution, while a Brookfield rotational viscometer (RV) captured viscosity–time dependencies. Mechanical performance (tensile properties, single-lap shear, and pull-off adhesion) was quantified using a universal testing machine (UTM) and an automatic adhesion tester (AAT). Bond failure mechanisms between EABCs and steel substrates were also analyzed. Figure 1 illustrates a flow chart of EABC preparation and characterization.

## 2. Results and Discussion

### 2.1. Rotational Viscosity–Time Characteristics

The rotational viscosity of epoxy asphalts increases with both temperature and time during curing. This results from molecular weight growth caused by the reaction between epoxide groups in epoxy oligomers and active hydrogen atoms in curing agents, culminating in three-dimensional crosslinked network formation [47,48,49]. This behavior fundamentally contrasts with neat bitumen. Additionally, viscosity–time characteristics are significantly influenced by multiple factors, including epoxy-to-bitumen ratios, epoxy resin/curing agent systems, stoichiometric ratios, and preparation procedures [12,50].

**Figure 2:**
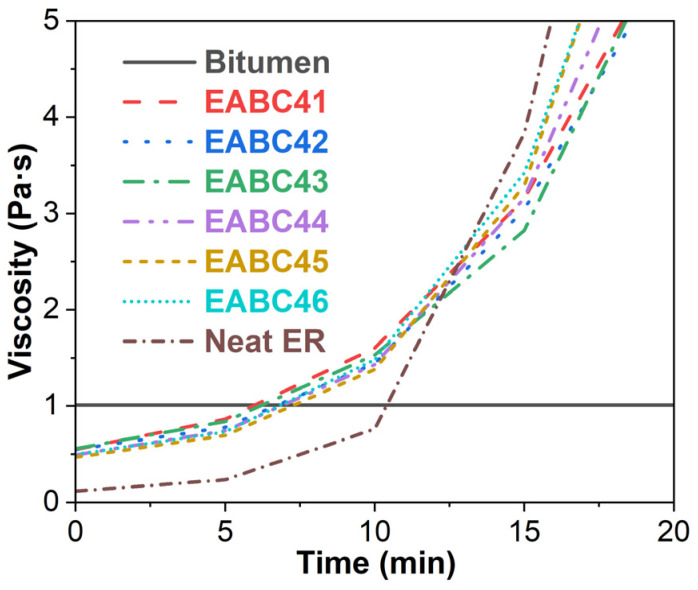
Viscosity versus time curves of bitumen, neat ER, and EABCs at 120 °C.

Figure 2 displays the viscosity–time profiles of bitumen, ER, and EABCs at 120 °C. While bitumen maintains a constant viscosity of 1.01 Pa·s at this temperature, the initial viscosity of neat ER (0.115 Pa·s) is substantially lower. As the curing reaction progresses, ER’s viscosity increases and surpasses bitumen’s viscosity after approximately 10 min. For EABCs, the initial viscosity lies between these two components due to combined material effects, and then increases progressively. After ~6 min, the EABCs’ viscosity exceeds bitumen’s, but remains below ER’s viscosity. At 13 min, all EABC formulations exhibit lower viscosities than neat ER and increase progressively with increasing ER concentration. According to GB/T30598-2014 [51], the time for EABCs to reach 5 Pa·s should exceed 10 min. As shown in Table 1, all EABCs meet this requirement. Moreover, increasing ER contents proportionally accelerates the attainment of 5 Pa·s in EABCs, as the bitumen’s diluting effect diminishes during the later-stage cure reaction between epoxide groups and active hydrogen atoms. This inversely correlates with allowable construction time at higher ER concentrations.

### 2.2. Phase Morphology Evolution

**Figure 3:**
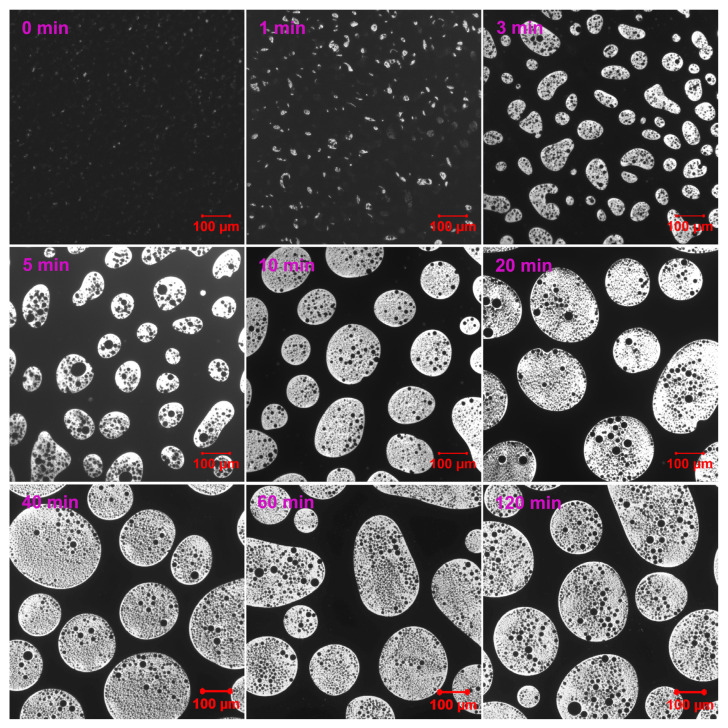
Reaction-induced phase evolution in EABC41 at 120 °C.

Figure 3 demonstrates the phase evolution of EABC with 41 vol.% ER (EABC41) at 120 °C. Rapid curing initially induces reaction-induced phase separation, evidenced by discrete white epoxy particles in the continuous black bitumen phase at *t* = 0 min [52]. During subsequent curing: (a) epoxy domains progressively coalesce and increase in size (a nucleation and growth [NG] phase separation mechanism [53]); (b) particle morphology transitions from irregular to spheroidal; and (c) bitumen sub-inclusions become encapsulated within epoxy droplets. This hierarchical “double emulsion” structure mirrors phenomena in both oil/water emulsions [54] and polymer blends [55,56].

**Figure 4:**
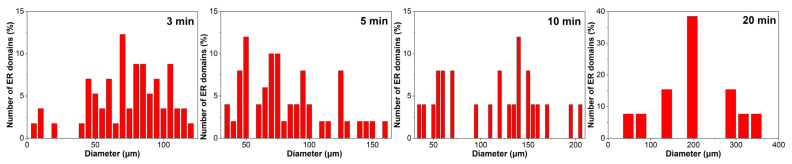
ER particle size distribution within the continuous bitumen phase of EABC41 during curing at 120 °C.

Figure 4 illustrates the particle size distribution of ER within the continuous bitumen phase of EABC41 during phase evolution at 120 °C. After 3 min of curing, the majority of ER particles are distributed in the 50–100 μm range. As the curing progresses, the particle size distribution expands to broader ranges: 50–150 μm at 5 min and 50–200 μm at 10 min. Following 20 min of curing, the particle size is concentrated predominantly at 200 μm.

Three LSCM micrographs were used to quantitatively analyze number-average and weight-average domain diameters (*D*_n_ and *D*_w_) using Image-Pro Plus 6.0, calculated with Equations (1) and (2) [57,58]:

(1) $$D_{n}=\frac{\Sigma n_{i}D_{i}}{\Sigma n_{i}}$$

(2) $$D_{w}=\frac{\Sigma n_{i}{D_{i}}^{2}}{\Sigma n_{i}D_{i}}$$

In these equations, *n*_i_ is the number of domains having a diameter *D*_i_.

The distribution of dispersed domains was calculated using the polydispersity index (PDI), which is the ratio of number-average diameter to weight-average diameter, interfacial area per unit volume (*a*), and interparticle distance (IPD) using the following Equations (3)–(5) [59,60].

(3) PDI=DwDn

(4) $$a=\frac{6\phi }{D_{n}}$$

where ϕ denotes the volume fraction of the dispersed phase.

(5) $$IPD=D_{n}\left[{\left(\frac{\Pi }{6\phi }\right)}^{\frac{1}{3}}\right]-1$$

As depicted in Table 2, prolonged curing time leads to increased ER domain size and interparticle distance, while reducing interfacial area through ER domain coalescence. Notably, this coalescence process exhibits minimal impact on the polydispersity index of EABC41.

**Figure 5:**
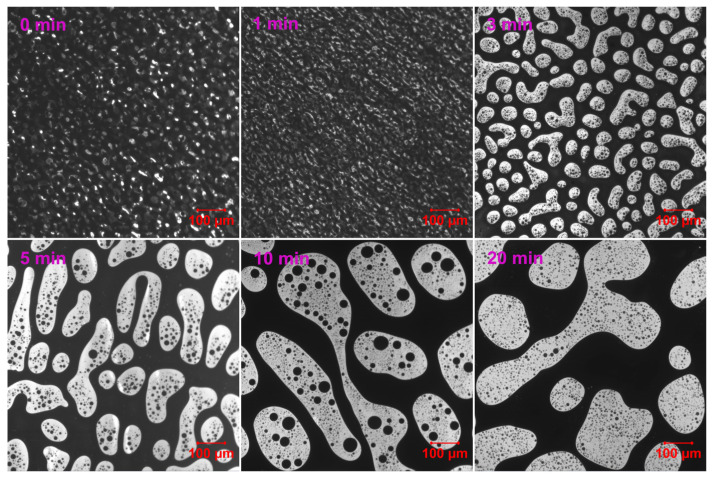
Reaction-induced phase evolution in EABC42 at 120 °C.

Figure 5 demonstrates that EABC with 42 vol.% ER (EABC42) initially exhibits larger epoxy particle sizes compared to EABC41. Remarkably, within just 1 min of curing at 120 °C, the epoxy particles coalesce into a secondary continuous phase within the bitumen matrix, indicating the development of a co-continuous morphology. Subsequently, after 3 min of curing, this co-continuous ER phase fragments into discrete droplets exhibiting irregular geometries. Following 20 min of curing at 120 °C, EABC42 develops epoxy domains exhibiting significantly greater shape irregularity compared to EABC41.

**Figure 6:**
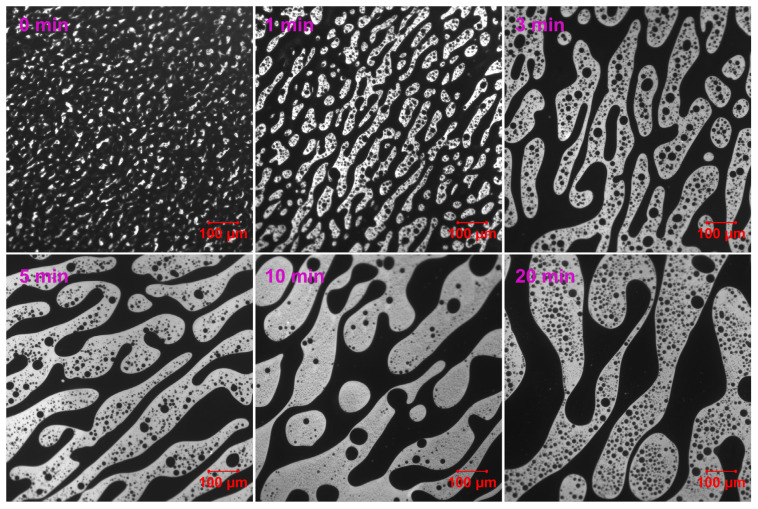
Reaction-induced phase evolution in EABC43 at 120 °C.

Compared to EABC42, EABC with 43 vol.% ER (EABC43) demonstrates enhanced phase contrast with significantly brighter ER domains during initial curing (Figure 6). Following 1 min of thermal treatment at 120 °C, the co-continuous phase in EABC43 propagates progressively with extended curing duration. Remarkably, this co-continuous architecture persists intact even after 20 min of curing.

**Figure 7:**
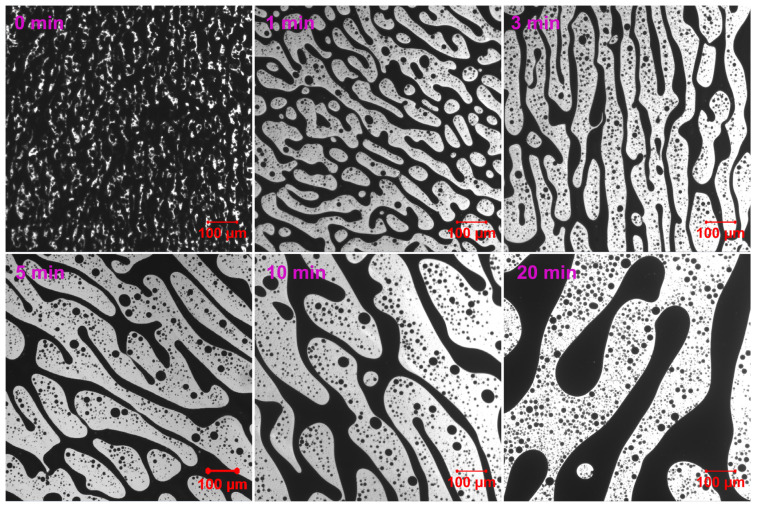
Reaction-induced phase evolution in EABC44 at 120 °C.

In contrast to EABC41–43, EABC with 44 vol.% ER (EABC44) develops a co-continuous morphology during initial curing at 120 °C (Figure 7). The co-continuous phase exhibits progressive expansion with prolonged curing duration. Notably, the domain size of co-continuous structures in EABC42–44 shows a positive correlation with increasing ER content.

**Figure 8:**
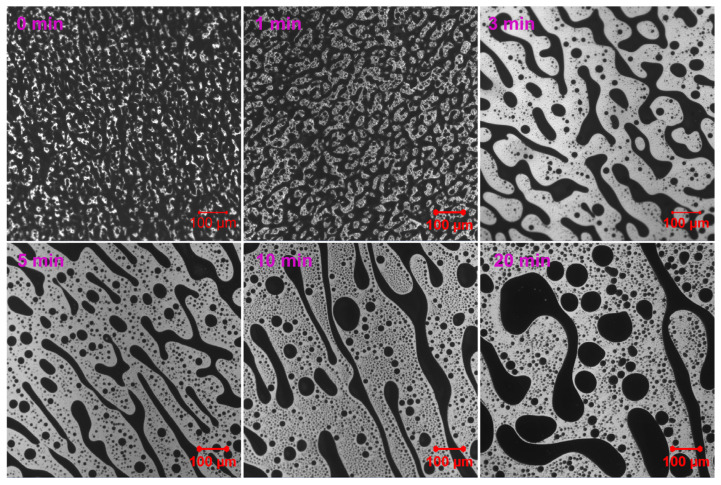
Reaction-induced phase evolution in EABC45 at 120 °C.

For EABC with 45 vol.% ER (EABC45), the characteristic dimensions of co-continuous structures after 1 min of curing at 120 °C are comparable to those observed in EABC42, as shown in Figure 8. Subsequently, mirroring the behavior of the EABC42–44 series, the epoxy constituents within these co-continuous domains undergo a progressive increase with extended curing duration. Notably, following 20 min of thermal curing, the morphological evolution of EABC45 progresses beyond co-continuous structures, resulting in the formation of numerous discrete bitumen domains dispersed throughout the continuous epoxy matrix. Interestingly, after 20 min of thermal curing at 120 °C, both EABC42 and EABC45 develop structurally analogous morphologies, albeit with inverted phase continuity: epoxy constitutes the dispersed phase in EABC42, while bitumen forms discrete domains within the continuous epoxy matrix in EABC45.

**Figure 9:**
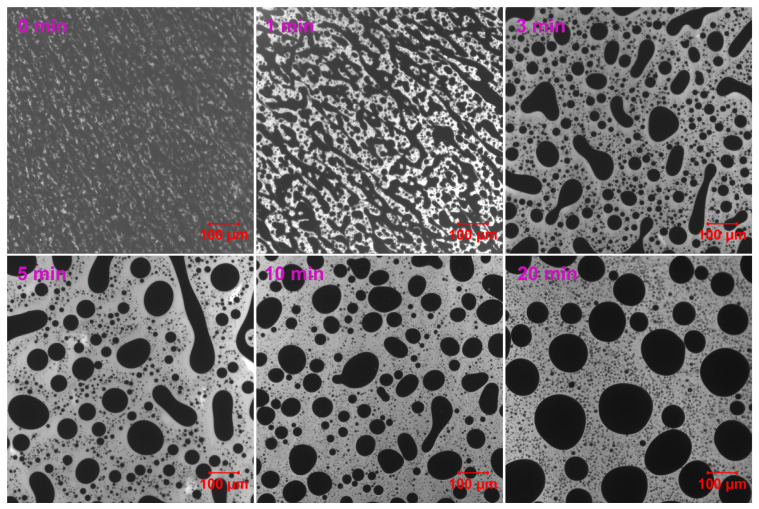
Reaction-induced phase evolution in EABC46 at 120 °C.

For EABC with 46 vol.% ER (EABC46), the co-continuous structures undergo phase inversion after 3 min of curing at 120 °C (Figure 9), transforming into discrete bitumen domains. These irregularly shaped bitumen domains subsequently evolve toward spherical morphology with prolonged curing.

**Figure 10:**
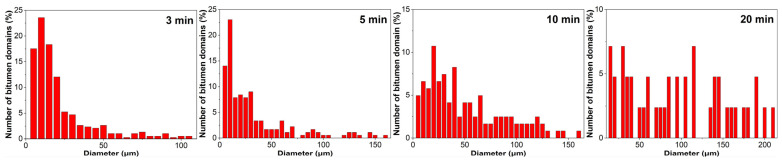
Particle size distribution of bitumen domains within EABC46 during curing at 120 °C.

Figure 10 characterizes the size distribution evolution of bitumen domains in EABC46 during thermal curing at 120 °C. At 3 and 10 min, the distributions are predominantly confined to smaller diameters (5–30 μm and 5–40 μm, respectively). With progressive curing, the distribution broadens toward larger diameters after 10 and 20 min, spanning 5–130 μm and 5–210 μm, respectively.

As quantified in Table 3, both the number-average diameter and interparticle distance of bitumen domains in EABC46 increase progressively with thermal curing at 120 °C. The weight-average diameter and interfacial area initially increase, plateau between 5 and 10 min, and then resume growth after 10 min. While the polydispersity index remains constant from 3 to 10 min, it increases significantly at 20 min, indicating enhanced heterogeneity in bitumen domain dispersion within the continuous epoxy matrix.

**Figure 11:**
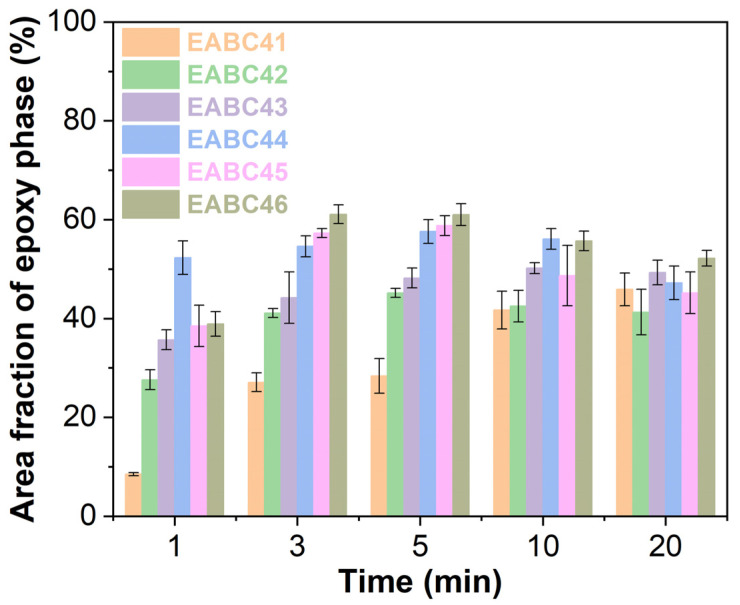
Area fractions of epoxy phases of EABCs during curing at 120 °C.

Figure 11 shows the evolution of epoxy phase area fractions in EABCs during curing at 120 °C. In epoxy-dominated and initial co-continuous structures (EABC41–43), the epoxy phase area fraction exhibits a continuous increase with curing time. For later co-continuous and bitumen-dominated morphologies (EABC44–46), the epoxy phase area fraction initially increases, reaching a maximum value at 5 min, after which it shows a slight decrease with further curing time.

### 2.3. Morphology of Cured EABCs

**Figure 12:**
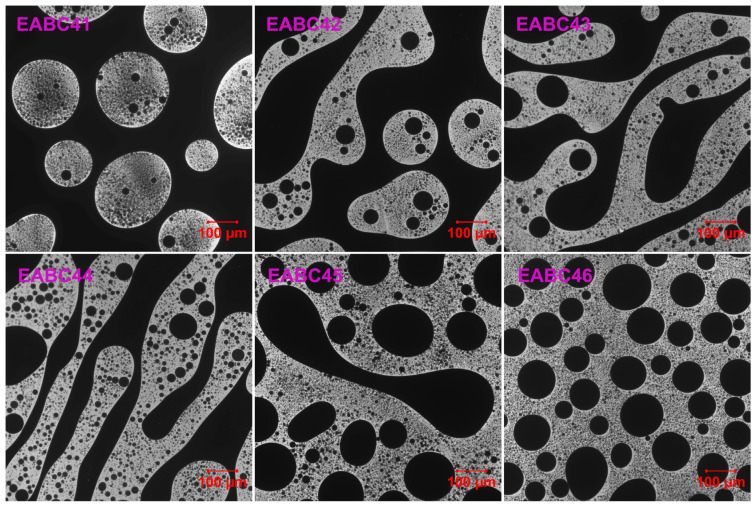
LSCM micrographs of cured EABCs.

Figure 12 presents LSCM images of EABCs cured at 120 °C for 4 h. Similar to morphologies observed after 20 min of curing (Section 2.2), three distinct phase-separated structures emerge with increasing ER concentration:

(1) Dispersed epoxy-rich domains within a continuous bitumen matrix (41–42 vol.% ER).
(2) Co-continuous phase structures (43–45 vol.% ER).
(3) Dispersed bitumen domains in a continuous epoxy matrix (46 vol.% ER).

In EABC42, where bitumen serves as the continuous matrix phase, larger epoxy domains with irregular morphology enhance mechanical properties, as discussed in Section 2.4. This behavior indicates that EABC42 represents a transitional structure between an epoxy-dominated and a co-continuous morphology.

**Figure 13:**
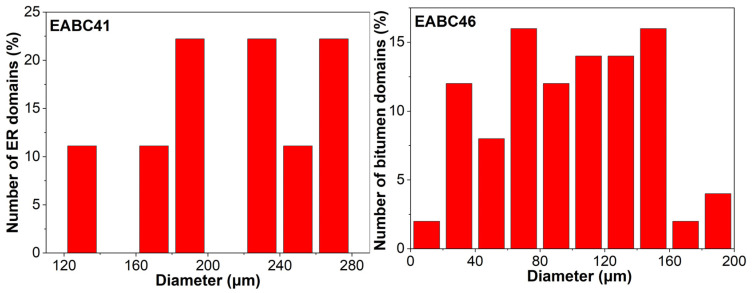
Particle size distribution of dispersed domains within cured EABC41 and EABC46.

Figure 13 compares domain size distributions between epoxy domains in cured EABC41 (bitumen-continuous matrix) and bitumen domains in cured EABC46 (epoxy-continuous matrix). Epoxy domains in the thermoplastic bitumen matrix range from 120 to 280 μm, while bitumen domains in the crosslinked epoxy matrix are significantly smaller (30–150 μm). This size reduction demonstrates that the crosslinked epoxy network more effectively constrains domain growth than the non-crosslinked bitumen matrix after phase inversion.

As quantified in Table 4, after phase inversion from epoxy-rich domains to bitumen-rich domains, the values of *D*_n_, *D*_w_, and IPD for the bitumen domains in EABC46 decrease to approximately half of those for the epoxy domains in EABC41. However, the interfacial area per unit volume increases by more than twofold. The PDI increases from 1.08 to 1.27, indicating that the dispersion of bitumen domains in EABC46 is more heterogeneous than that of epoxy domains in EABC41 due to suppressed coalescence kinetics resulting from epoxy-network-imposed confinement on bitumen domains.

**Figure 14:**
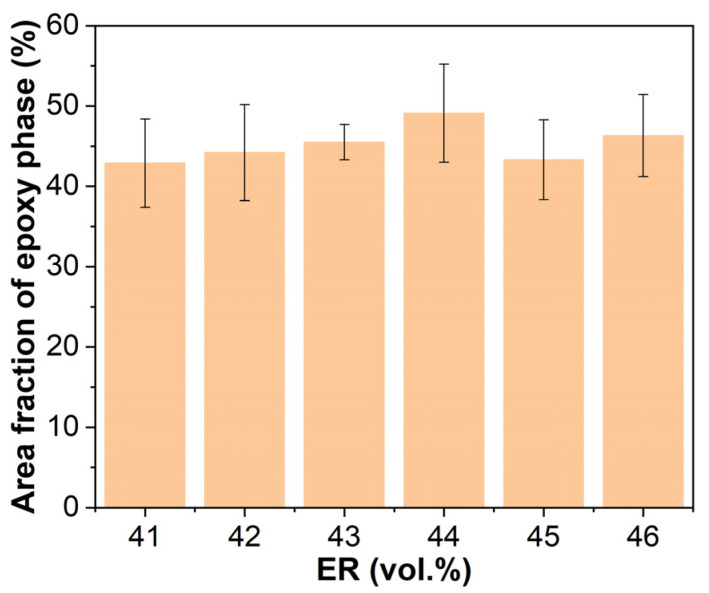
Area fractions of epoxy phase in cured EABCs.

Figure 14 illustrates the epoxy phase area fractions in cured EABCs. The area fraction initially rises with increasing ER concentration from 41 to 44 vol.%, then declines at 45 vol.% before showing a minor rebound at 46 vol.%.

### 2.4. Tensile Properties

**Figure 15:**
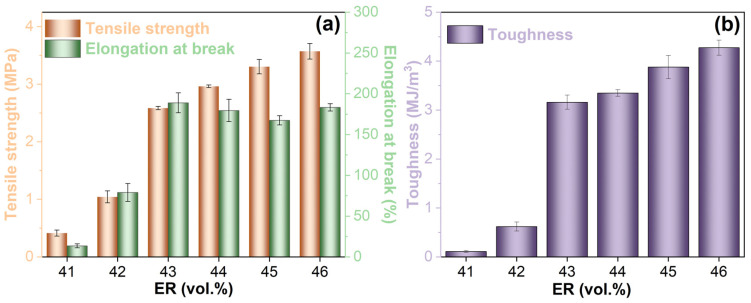
Tensile strength, elongation at break (**a**), and toughness (**b**) of cured EABCs.

Figure 15 demonstrates the tensile performance characteristics of cured EABC materials. In systems with continuous bitumen phases (EABC41 and EABC42), tensile properties exhibit significant improvement with increasing ER concentration. This enhancement is attributed to the formation of larger epoxy domains with irregular morphology and the substantial mechanical property differential between the bitumen matrix and cured epoxy resin phases. The phase transition from bitumen-continuous to co-continuous structure yields particularly notable improvements in tensile performance. Relative to EABC41 and EABC42, EABC43 displays 148% and 524%, 139% and 1298%, and 409% and 2732% increases in tensile strength, elongation at break, and toughness, respectively. For co-continuous EABC systems (EABC43–EABC45), tensile strength and toughness increase progressively with ER concentration, while elongation at break exhibits a decreasing trend. The microstructural transition to a continuous epoxy phase further enhances tensile strength as well as elongation at break and toughness. Notably, the increase in tensile strength and toughness observed either within co-continuous structures or during the transition to an epoxy-continuous structure is significantly smaller than that accompanying the shift from bitumen-continuous to co-continuous microstructure.

### 2.5. Pull-Off Adhesion Strength

**Figure 16:**
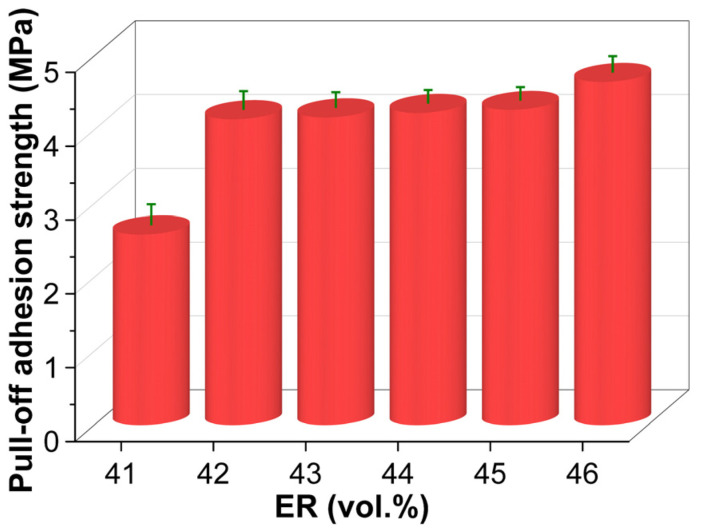
Pull-off adhesion strength of cured EABCs.

Figure 16 shows the pull-off adhesion strength of cured EABC materials. Similar to tensile properties, the bitumen-continuous systems exhibit a 60% increase in pull-off adhesion strength when the ER content rises from 41 to 42 vol.%. However, the microstructural transition from a bitumen-continuous to a co-continuous structure does not yield a significant improvement. A gradual enhancement in pull-off adhesion strength is observed with increasing ER concentration across the full phase transition spectrum, from a bitumen-continuous to a co-continuous and then to an epoxy-continuous structure (EABC42–EABC46). Notably, compared to EABC41, the pull-off adhesion strengths of EABC43 and EABC46 are significantly higher by 61% and 80%, respectively.

**Figure 17:**
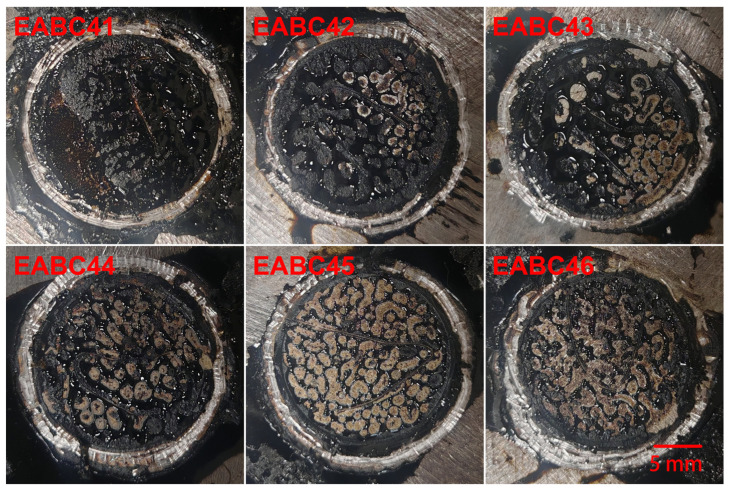
Failure surfaces of steel adherends after pull-off adhesion testing.

Figure 17 shows the failure surfaces of steel substrates after pull-off adhesion testing. Adhesively bonded joints exhibit two primary failure modes: adhesive failure at the adhesive/adherend interface and cohesive failure within the adhesive or adherend bulk [61]. Most failures involve a combination of both modes [62]. While EABC41 undergoes purely cohesive failure, other EABC systems exhibit mixed-mode failures. Although cohesive failure is generally preferable [63], EABC41, featuring spherical epoxy domains, exhibits the lowest pull-off adhesion strength due to the inherently weaker bonding capability of its continuous bitumen matrix compared to the continuous epoxy matrix.

**Figure 18:**
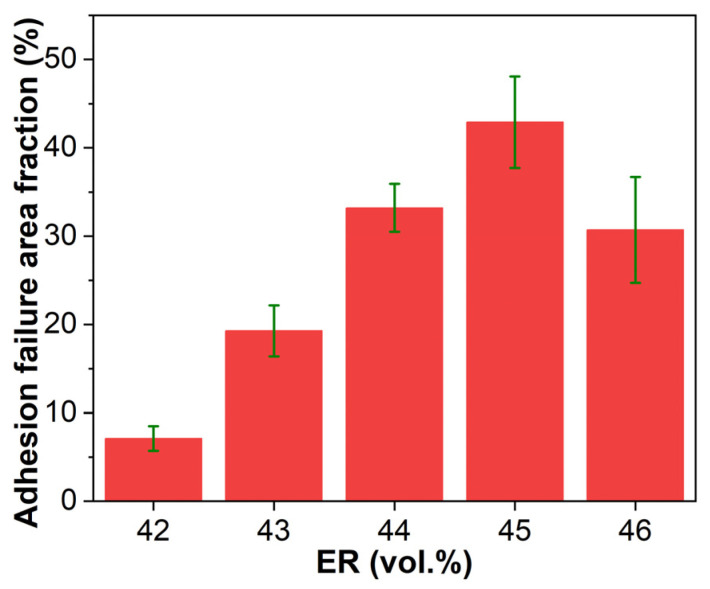
Adhesive failure area fractions on steel adherends after pull-off adhesion testing.

Figure 18 presents the adhesive failure area fractions on steel adherends after pull-off adhesion testing. For EABCs with bitumen-continuous through co-continuous structures (EABC42–EABC45), the adhesive failure area fraction increases with ER concentration due to the expansion of continuous epoxy components, which have superior bonding capability compared to bitumen. However, EABC46 with a continuous epoxy phase exhibits a decreased adhesive failure area, reflecting increased cohesion failure. Consequently, EABC46 achieves the highest pull-off adhesion strength among all systems, measuring 80% higher than EABC41 with a continuous bitumen phase.

### 2.6. Single-Lap Shear Strength

**Figure 19:**
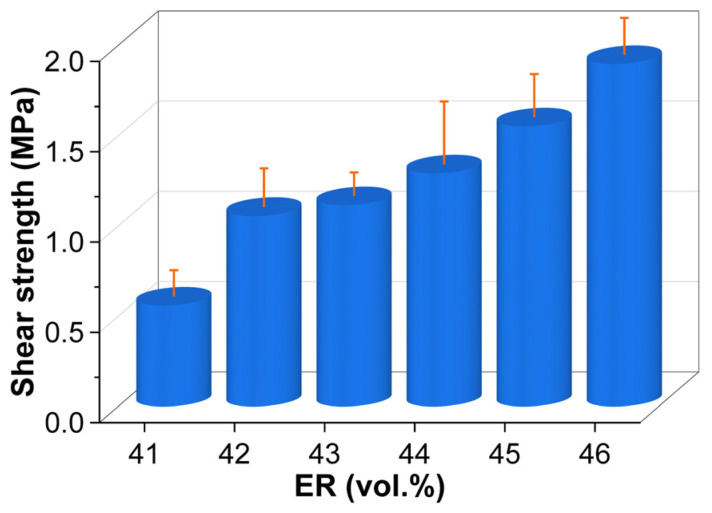
Single-lap shear strength of cured EABCs.

Figure 19 illustrates the single-lap shear strength of cured EABCs. Like pull-off adhesion strength, shear strength exhibits a dramatic 89% increase when ER concentration rises from 41 vol.% to 42 vol.%. Beyond 42 vol.%, shear strength progresses steadily with increasing ER concentration, though the rate of growth between consecutive concentrations exceeds that of pull-off adhesion strength (Figure 16). Compared to EABC41, EABC43 and EABC46 show 99% and 240% higher shear strength.

**Figure 20:**
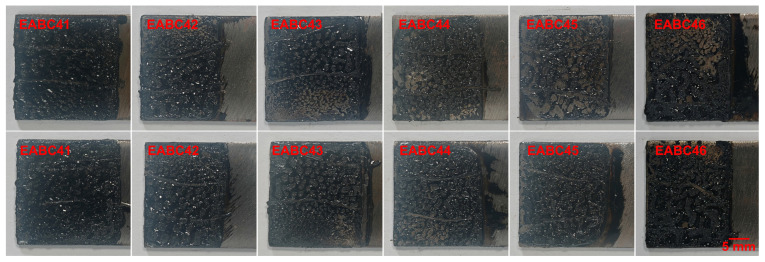
Representative failure surfaces of mated steel adherends after single-lap shear testing.

As shown in Figure 20, EABC specimens demonstrate failure modes consistent with those observed in pull-off adhesion testing (Figure 17) following single-lap shear testing. Specifically, EABC41 undergoes cohesive failure, whereas all other systems display mixed cohesive–adhesive failure mechanisms.

**Figure 21:**
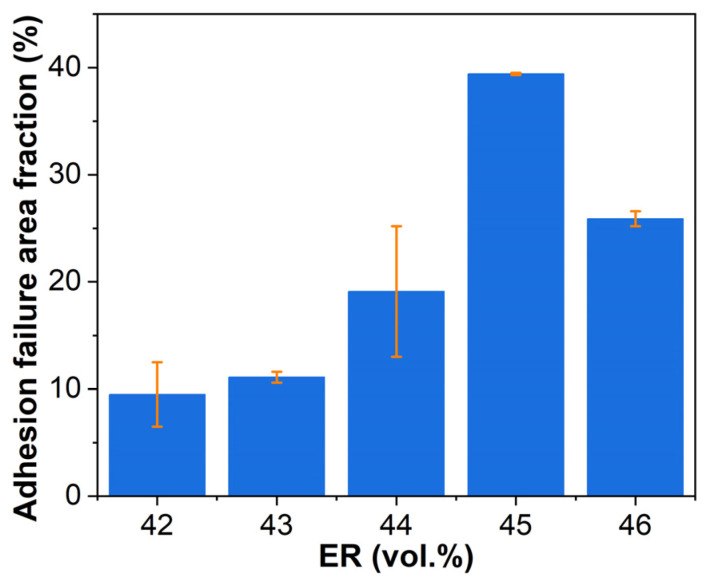
Adhesive failure area fractions on steel adherends after single-lap shear testing.

Figure 21 presents the adhesive failure area fractions on steel adherends after single-lap shear testing. EABC systems exhibit trends analogous to those in pull-off adhesion testing (Figure 18), demonstrating consistent failure progression.

## 3. Materials and Methods

### 3.1. Materials

Bitumen was sourced from China Offshore Bitumen (Taizhou) Co., Ltd. (Taizhou, China). Key characteristics of the bitumen are summarized in Table 5. The acid-based curing agent was independently formulated in our laboratory. Bisphenol A-based epoxy oligomers (epoxide equivalent weight: 192 g/eq) were procured from Nantong Xingchen Synthetic Material Co., Ltd. (Nantong, China).

### 3.2. EABC Preparation

Bitumen and the curing agent were preheated to 120 °C to ensure uniform viscosity. The curing agent was introduced into the bitumen in a 250 mL beaker, followed by 20 min of heating in a 120 °C oven. Subsequently, epoxy oligomers (preheated to 60 °C) were incorporated into the mixture and stirred at 200 rpm for 1 min at 120 °C. The uncured sample was then immediately transferred into a Teflon mold and cured at 120 °C for 4 h. The mass ratio of epoxy oligomers and curing agents at a stoichiometry of 1.0 was maintained at 1:2.5. The density of cured epoxy resin was 1.08 g/cm^3^. The resulting EABCs contained epoxy resin contents of 41–46 vol.% (43–48 wt%).

### 3.3. Methods

#### 3.3.1. Phase-Separated Morphology Observations

The phase structure evolution of uncured EABCs and the final morphology of cured EABCs were characterized using a Zeiss LSM710 LSCM (Jena, Germany). A 488 nm laser excitation source (argon ion) was employed for imaging.

Sample preparation for phase evolution analysis:

(1) Uncured EABC droplets were deposited onto glass slides preheated to 120 °C on a hot stage.
(2) Cover slips were applied and lightly compressed to ensure uniform film formation.
(3) Two slides (0 min curing) were immediately quenched in a cryogenic conditioner.
(4) Remaining slides were cured in a 120 °C oven and quenched at intervals of 1, 3, 5, 10, 20, and 240 min.

The sample cured for 240 min at 120 °C was designated as the fully cured EABC for final morphology evaluation. For the initial curing stage, some slides were quenched and frozen in a conditioner, while other slides were further cured at 120 °C and then frozen. Finally, all slides were taken out and observed using the LSCM.

#### 3.3.2. Rotational Viscometry

Following ASTM D4402 [67], viscosity evolution was monitored using a Changji NDJ-1 Brookfield rotational viscometer (Shanghai, China). Spindle #18 rotated at 0.83 s^−1^ at 120 °C, with measurements recorded continuously until the viscosity reached 5000 mPa·s.

#### 3.3.3. Tensile Testing

Tensile properties were analyzed using a Shimadzu AGX-10kNVD universal testing machine (Kyoto, Japan) in accordance with ASTM D638 Type V specifications [70]. Dumbbell-shaped specimens were tested at 200 mm/min crosshead speed and room temperature with a 10 kN load cell. Five replicates were tested per formulation.

#### 3.3.4. Pull-Off Adhesion Testing

Pull-off adhesion strength was measured according to ASTM D4541-22 [71] using a PosiTest AT-A portable automatic adhesion tester (DeFelsko, Ogdensburg, NY, USA). Uncured EABC specimens were applied to polished Q345D steel substrates (150 × 150 × 10 mm^3^) using 0.2 mm copper wire spacers to control film thickness. After curing at 120 °C for 4 h, five 20 mm diameter dollies were bonded to the EABC surfaces. Test areas were isolated by circumferential scoring around each dolly using a core drill. The tester subsequently applied perpendicular tensile loading at 0.7 MPa/s until failure at room temperature.

#### 3.3.5. Single-Lap Shear Testing

Shear strength was determined via single-lap shear testing per ASTM D1002 [72]. Uncured EABC was applied to the bonding area of stainless steel substrates (100 × 25 × 2 mm^3^). The plates were subsequently overlapped by 12.5 mm, sandwiching the adhesive layer, then cured at 120 °C for 4 h. After cooling to ambient temperature, specimens were tension-tested at 50 mm/min crosshead speed using a Shimadzu AGX-10kNVD universal testing machine (Kyoto, Japan) in tension mode at a strain rate of 10 mm/min. Five replicates were evaluated per formation.

## 4. Conclusions

Epoxy asphalt bond coats with three distinct microstructures (bitumen-continuous, co-continuous, and epoxy-continuous) were systematically prepared. The rotational viscosity–time profiles, phase morphology evolution during curing, and final phase-separated morphology were comprehensively investigated. Mechanical properties were evaluated through tensile tests, pull-off adhesion measurements, and single-lap shear testing. The principal findings are summarized as follows:

- The rotational viscosity of EABCs demonstrates a progressive increase with increasing ER concentration at the later curing stage, resulting from the combined effects of bitumen’s static viscosity and the epoxy’s dynamic viscosity.
- The phase microstructure development follows three distinct stages: epoxy-dispersed morphology in a continuous bitumen matrix via a nucleation and growth mechanism (41–42 vol.% ER), co-continuous morphology (43–45 vol.% ER), and bitumen-dispersed morphology in continuous epoxy networks (46 vol.% ER). Notably, EABC42 represents a transitional structure between epoxy-dispersed and co-continuous configurations.
- Cured EABC46 exhibits substantially reduced bitumen domain dimension (compared to cured EABC41) due to the constraining effect of crosslinked epoxy networks.
- Dramatic property enhancement occurs during the epoxy-dispersed to co-continuous transition (e.g., tensile strength: 0.41–1.04 MPa; pull-off adhesion strength: 2.58–4.15 MPa; single-lap shear strength: 0.56–1.06 MPa), while only incremental improvements are observed within the co-continuous region and during subsequent transition to bitumen-dispersed morphology (e.g., tensile strength: 2.59–2.96 MPa; pull-off adhesion strength: 4.17–4.23 MPa; single-lap shear strength: 1.11–1.29 MPa).

Notably, the effects of polymer modifiers (such as toughening agents) and nanofillers on the morphological evolution and mechanical performance of co-continuous epoxy asphalts merit systematic investigation in subsequent research.

## Figures and Tables

**Table 1:** Time to reach 5 Pa·s for neat ER and EABCs at 120 °C.

ER (vol.%)	41	42	43	44	45	46	100
**Time to reach 5** **Pa·** **s (min)**	19.1	18.9	18.8	18.0	17.1	17.1	16.1

**Table 2:** Morphology parameters of EABC41 during phase evolution at 120 °C.

Time (min)	*D*_n_ (μm)	*D*_w_ (μm)	PDI	*a* (μm^−1^)	IPD (μm)
3	65.7 (±7.6)	77.7 (±6.2)	1.18	0.038	70.2
5	89.6 (±5.6)	102.2 (±5.8)	1.14	0.028	96.1
10	151.5 (±16.3)	178.3 (±15.7)	1.18	0.016	163.2
20	204.1 (±5.8)	240.6 (±18.1)	1.18	0.012	220.2

**Table 3:** Morphology parameters of EABC46 during phase evolution at 120 °C.

Time (min)	*D*_n_ (μm)	*D*_w_ (μm)	PDI	a (μm^−1^)	IPD (μm)
3	31.4 (±0.4)	52.6 (±1.0)	1.18	0.088	31.8
5	52.7 (±2.4)	77.4 (±1.8)	1.14	0.053	54.0
10	52.9 (±3.7)	71.1 (±7.6)	1.18	0.052	54.2
20	101.7 (±8.4)	132.2 (±2.2)	1.30	0.027	105.1

**Table 4:** Morphology parameters of epoxy domains in cured EABC41 and bitumen domains in EABC46 at 120 °C.

Sample	*D*_n_ (μm)	*D*_w_ (μm)	PDI	A (μm^−1^)	IPD (μm)
EABC41	231.4 (±29.1)	250.2 (±20.6)	1.08	0.011	250.0
EABC46	101.1 (±3.1)	128.5 (±13.4)	1.27	0.027	104.5

**Table 5:** Physicochemical characteristics of bitumen.

Properties	Standard	Value
Physical properties		
Penetration (25 °C, 0.1 mm)	ASTM D5 [64]	73.0
Ductility (10 °C, cm)	ASTM D113 [65]	15.8
Softening point (°C)	ASTM D36 [66]	48.2
Viscosity (60 °C, Pa·s)	ASTM D4402 [67]	173.0
Density (25 °C, g/cm^3^)	ASTM D8188 [68]	1.01
Chemical components		
Saturates (%)	ASTM D4124 [69]	20.0
Aromatics (%)		31.5
Resins (%)		37.1
Asphaltenes (%)		6.8

## Data Availability

All data are available in the manuscript.
